# Magnetic isotope and magnetic field effects on the DNA synthesis

**DOI:** 10.1093/nar/gkt537

**Published:** 2013-07-13

**Authors:** Anatoly L. Buchachenko, Alexei P. Orlov, Dmitry A. Kuznetsov, Natalia N. Breslavskaya

**Affiliations:** ^1^Institute of Chemical Physics, Russian Academy of Sciences, 119991 Moscow, Russian Federation, ^2^Institute of Problems of Chemical Physics, Russian Academy of Sciences, 142432 Chernogolovka, Russian Federation, ^3^Moscow State University, 119992 Moscow, Russian Federation, ^4^Russian National Medical University, 117997 Moscow, Russian Federation and ^5^Institute of General and Inorganic Chemistry, Russian Academy of Sciences, 119991 Moscow, Russian Federation

## Abstract

Magnetic isotope and magnetic field effects on the rate of DNA synthesis catalysed by polymerases β with isotopic ions ^24^Mg^2+^, ^25^Mg^2+^ and ^26^Mg^2+^ in the catalytic sites were detected. No difference in enzymatic activity was found between polymerases β carrying ^24^Mg^2+^ and ^26^Mg^2+^ ions with spinless, non-magnetic nuclei ^24^Mg and ^26^Mg. However, ^25^Mg^2+^ ions with magnetic nucleus ^25^Mg were shown to suppress enzymatic activity by two to three times with respect to the enzymatic activity of polymerases β with ^24^Mg^2+^ and ^26^Mg^2+^ ions. Such an isotopic dependence directly indicates that in the DNA synthesis magnetic mass-independent isotope effect functions. Similar effect is exhibited by polymerases β with Zn^2+^ ions carrying magnetic ^67^Zn and non-magnetic ^64^Zn nuclei, respectively. A new, ion–radical mechanism of the DNA synthesis is suggested to explain these effects. Magnetic field dependence of the magnesium-catalysed DNA synthesis is in a perfect agreement with the proposed ion–radical mechanism. It is pointed out that the magnetic isotope and magnetic field effects may be used for medicinal purposes (trans-cranial magnetic treatment of cognitive deceases, cell proliferation, control of the cancer cells, etc).

## INTRODUCTION

DNA polymerases are known to accomplish DNA replication incorporating nucleotides into the DNA strand by two-magnesium-ion mechanism (1,2). One of the ions is tightly bound with pyrophosphate group of the incoming nucleotide and assists the departure of this group from the catalytic site after the insertion (incorporation) of the nucleotide into the growing DNA molecule is completed. The other ion coordinates 3′O atom of the DNA and P_α_ atom of the incoming nucleotide to facilitate nucleophilic in-line attack of 3′O on the P_α_ atom; it is supposed to function as a catalytic ion. Different possible mechanisms for the addition reaction catalysed by DNA polymerase β (pol β) were extensively explored by combined technique of quantum/molecular mechanics (QM/MM) (3–6). The common motif of all diversity of the considered mechanisms is a nucleophilic attachment of 3′O atom to the P_α_ atom catalysed by Mg^2+^ ion to form P–O chemical bond. This elementary reaction is almost identical to that for the ATP synthesis by ATP synthase or kinases; both these reactions are supposed to be nucleophilic.

The ATP synthesis was recently shown to exhibit magnetic isotope effect: the ATP-generating activity of enzymes loaded with Mg^2+^ ions containing magnetic nuclei ^25^Mg was shown to be two to three times higher than the activity of enzymes with non-magnetic spinless nuclei ^24^Mg and ^26^Mg (7–11). An identity of the nucleophilic key steps in these two processes, the addition of phosphate group to ADP and incorporation of nucleotide into the growing DNA strand, stimulated us to search for the influence of magnesium isotopes on the latter. For this purpose, we have measured enzymatic activity of the pol β in the presence of magnesium ions ^24^Mg^2+^, ^25^Mg^2+^ and ^26^Mg^2+^. The activity was found to strongly depend on the nuclear magnetic moment of magnesium, the result which discloses a new, incompatible with traditional, insight into the chemistry of the DNA synthesis both in replication and repairing processes. This conclusion is also confirmed by observation of the magnetic field effect and zinc isotope effect on the pol β activity.

## MATERIALS AND METHODS

### Materials

It is extremely important to control contamination of the used magnesium and zinc isotopes. As an example we would address to (12); in this study a large amount of contaminating Fe^3+^ (or Fe^2+^) ions in magnesium chloride have prevented detection of the magnetic effects in enzymatic ATP synthesis. In our samples, the traces of metals and other elements were detected by sparking mass spectroscopy (JEOL spectrometer JMS-01-BM2 with double focusing) and did not exceed 40 ppm even for the most dangerous contaminator, Fe ions (Table 1).

**Table 1.** The main impurities (in ppm)

	^24^MgO	^25^MgO	^26^MgO	*MgO
Mn	0.8	<0.1	2.0	0.07
Fe	30.0	5.0	40.0	0.3
Co	<0.05	0.2	<0.05	<2.0
Ni	<0.05	3.0	3.0	<3.0
Cu	30.0	3.0	7.0	<3.0
Zn	15.0	8.0	7.0	<0.6

No correlation between the impurity contents in the different magnesium oxides and their enzymatic activity is exhibited; it means that the activity is controlled by magnesium itself as a basic substance rather than by traces of other elements. Isotope-containing MgCl_2_ samples were obtained using treatment of magnesium oxides ^24^MgO, ^25^MgO, ^26^MgO and *MgO with analytically pure HCl (*Mg means magnesium with natural abundance of the three Mg isotopes).

### Measurement of the pol β activity

The measurements of the catalytic activity of isolated from HL-60 human myeloid leukaemia cells and purified enzyme DNA pol β were carried out using a slightly modified method proposed by (13) for 0.15-ml incubation mixtures consisting of 50 mM Tris–HCl (pH 8.0)/8.0 mM dithiothreitol/15% glycerol (v/v)/27 µg DNA, calf thymus/50 µg each of dATP, dCTP, dTTP, dGTP/0.1 µM [^3^H]dTTP, not less than 4.0 Ci/mmol/150 mM NaCl. Concentrations of MgCl_2_ were varied within a 0.2–50.0 mM range. These samples were first pre-incubated at 37°C for 60 min. Then 5.0–7.5 µg of pure enzyme was added to each one of these samples, and they were incubated at +37°C for 60 min longer. The ice cold incubation samples (0°C, 60 min pre-incubation) as well as the trypsin-treated samples (15 µg/ml trypsin, Sigma, USA, 37°C, 60 min) were taken for controls. Once the samples incubation completed, a final concentration of NaCl in each one of them was adjusted to 1.0 M followed by their placement into the ice bath (0°C). Thirty minutes later, the samples were passed through the Millipore FG600 membranes (0.2–0.4-µm-pore diameter, Millipore, France), and the resulted filtrates were treated with an ice-cold 2-methylenepentane-2,4-diol making the final concentration of the latter equal to 16.0% (v/v). The solutions obtained were subjected to quantitative extraction of the DNA using an AccuPrep Genomic DNA Extraction Kit (Bioneer Corp., Korea) as it has been described by (14) and then modified by (15). The DNA extract aliquots were used further for electrophoretic determination of the pol β-directed DNA chain sizes (16) as well as for total [^3^H]-radioactivity values in a standard dioxane liquid scintillation system (Wallac 2200LX LS Counter, Wallac OY, Finland). The pol β-specific catalytic activity values were expressed in amounts of the 1.0 min-synthesized nascent DNA radioactivity (i.e., [^3^H]TTP incorporation level) corrected for 1.0 mg pure enzyme protein, [^3^H] counts/min/mg pol β; it is further conventionally signified as a rate of DNA replication.

The protein ultra-micro amounts measurements were carried out according to (17). They were performed in diluted water solutions as described by (18). To find out a well-known β-specific resistance of the enzyme purified to both *N*-ethyl-melamide (0.5 mM, Sigma, USA) and Aphidicolin (5.0 µg/ml, Fluka, Switzerland), a special series of tests were conducted according to (13). These inhibitors were added to samples before the pre-incubation launched. For the same reason of β-type activity evidence, 200 mM KCl concentration has been adjusted in a separate control pre-incubation sample tests (13).

## RESULTS

### Magnesium isotope effect: ^25^Mg versus *Mg

**Figure 1.**
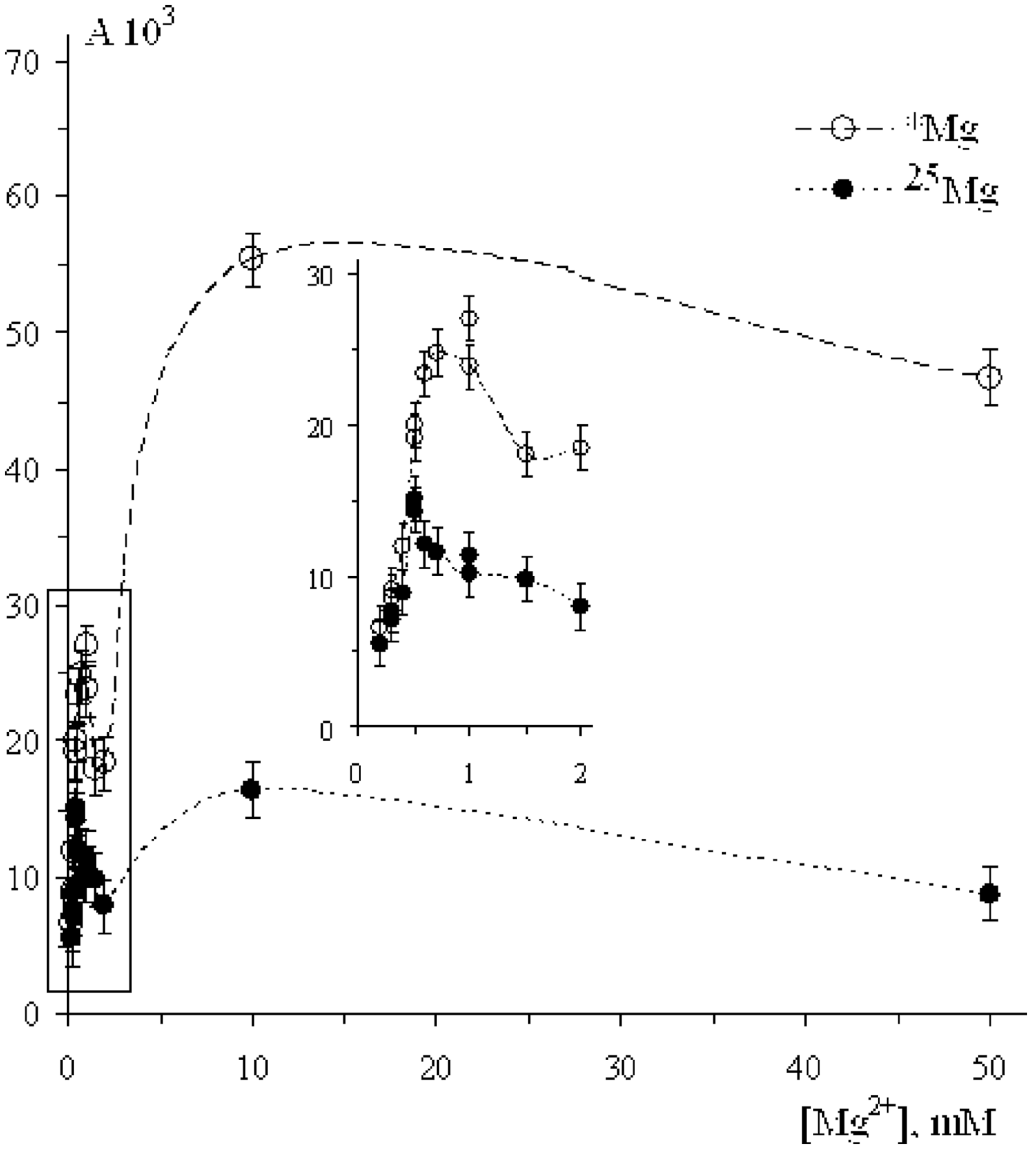
The rate of the DNA synthesis as a function of Mg^2+^ ion concentration. Tritium radioactivity A is measured as the number of counts/min/mg of DNA. The content of ^25^Mg in Mg^2+^ ions is 86.8%.

In this series of experiments, we have measured pol β activity in the presence of magnesium ions *Mg^2+^ with natural isotope composition (79% of ^24^Mg, 10% of ^25^Mg, 11% of ^26^Mg) and with ^25^Mg^2+^ ions (the content of ^25^Mg is 86.8%). Figure1 shows the yield of tritium-labelled DNA, i.e. the quantity of tritium labelled nucleotides TTP incorporated into the DNA strand in 1 min (the rate of DNA synthesis) as a function of magnesium ion concentration. It exhibits two remarkable properties. First, there are two maxima in the concentration dependence, first is in the region 0.5–1.0 mM (see insert in Figure 1), the other is placed at the concentration by order of magnitude higher, in the region 10–20 mM. Second, there is large isotope effect on the DNA synthesis: ^25^Mg^2+^ ions strongly suppress nucleotide incorporation into the DNA molecule at the ion concentrations ≥0.5 mM.

In the range 0.5–1.0 mM, its magnitude is ∼2; at the concentrations ≥2 mM, it is even larger and reaches magnitudes of 3–5. These high concentrations, of course, strongly exceed physiological ones; however, such a huge isotope effect is intriguing as a chemical phenomenon. Note that at low concentrations of ions (≤0.5 mM), DNA synthesis does occur, but it does not reveal isotope effect.

### ^25^Mg versus ^24^Mg

**Figure 2.**
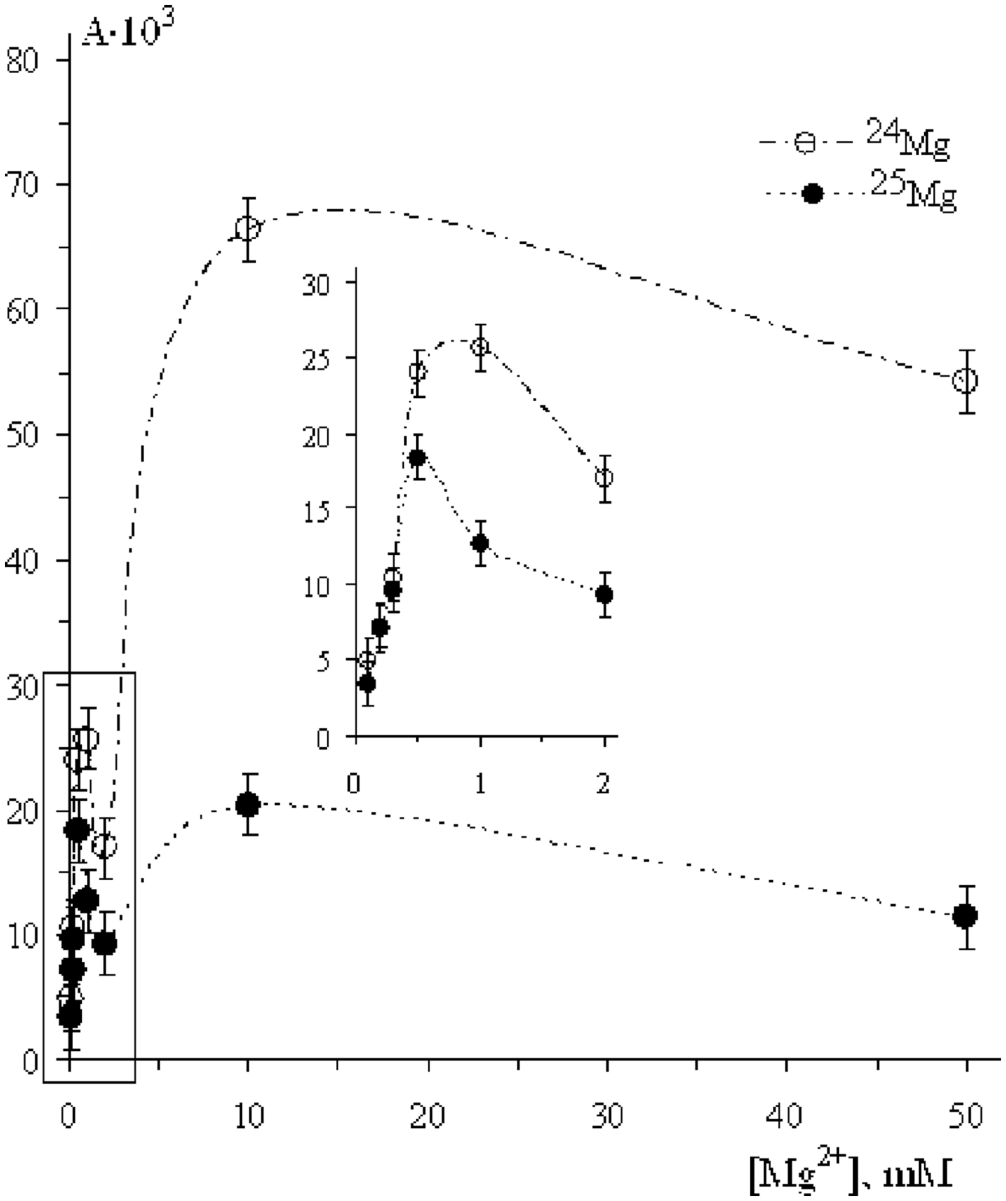
The rate of the DNA synthesis as a function of Mg^2+^ ion concentration. Tritium radioactivity A is measured as the number of counts/min/mg of DNA. The contents of ^25^Mg and ^24^Mg in Mg^2+^ ions are 86.8 and 98.6%, respectively.

In another series of experiments, highly enriched magnesium isotopes ^24^Mg (98.6%) and ^25^Mg (86.8%) were used. The activities A(^24^Mg) and A(^25^Mg), again measured as tritium radioactivity of [^3^H]TTP incorporated into the DNA, are shown in Figure 2 as a function of magnesium ion concentration.

At low concentrations of Mg^2+^ ions (they are in the range of physiological concentrations in cells), isotope effect is ignorable. The increasing of the ion concentration is accompanied simultaneously by increasing of the pol β activity and isotope effect. Both magnitudes reach maximum at the concentration about 1 mM and then both decrease. However at the concentrations >2 mM, pol β activity again strongly increases reaching the second maximum at the concentration in the region 10–20 mM.

### ^25^Mg versus ^26^Mg

**Figure 3.**
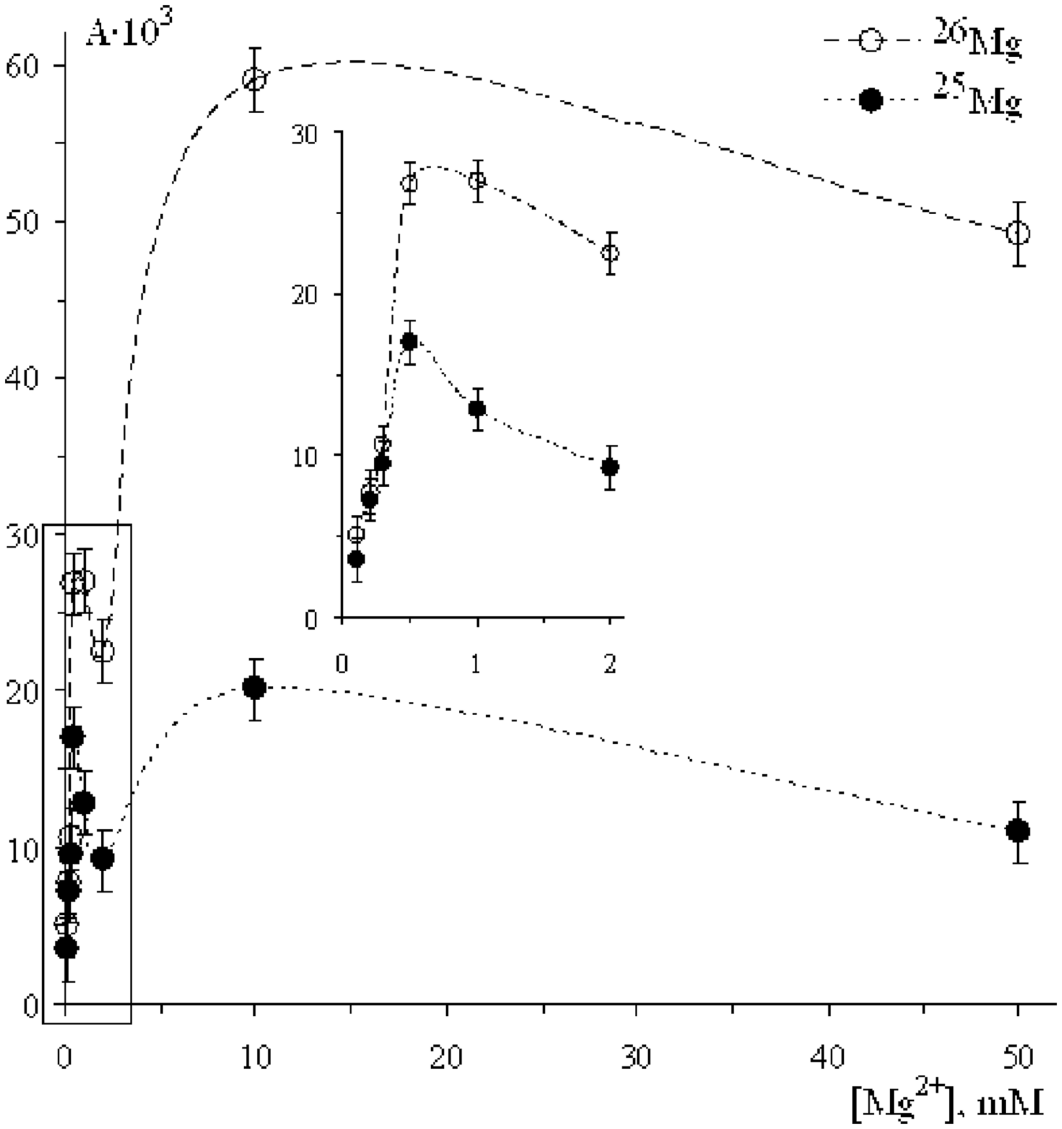
The rate of the DNA synthesis as a function of Mg^2+^ ion concentration. Tritium radioactivity A is measured as the number of counts/min/mg of DNA. The contents of ^25^Mg and ^26^Mg in Mg^2+^ ions are 86.8 and 98.4%, respectively.

In this series of experiments, magnesium isotopes ^26^Mg (98.4%) and ^25^Mg (86.8%) were used. The activities A(^26^Mg) and A(^25^Mg) measured as tritium radioactivity of [^3^H]TTP incorporated into the DNA are shown in Figure 3 as a function of magnesium ion concentration. Both concentration dependence and isotope effect exhibit behaviour almost identical to that shown in Figure 1 and Figure 2: the same two maxima, the same magnetic isotope effect.

### Zinc isotope effect: ^67^Zn versus ^64^Zn

**Figure 4.**
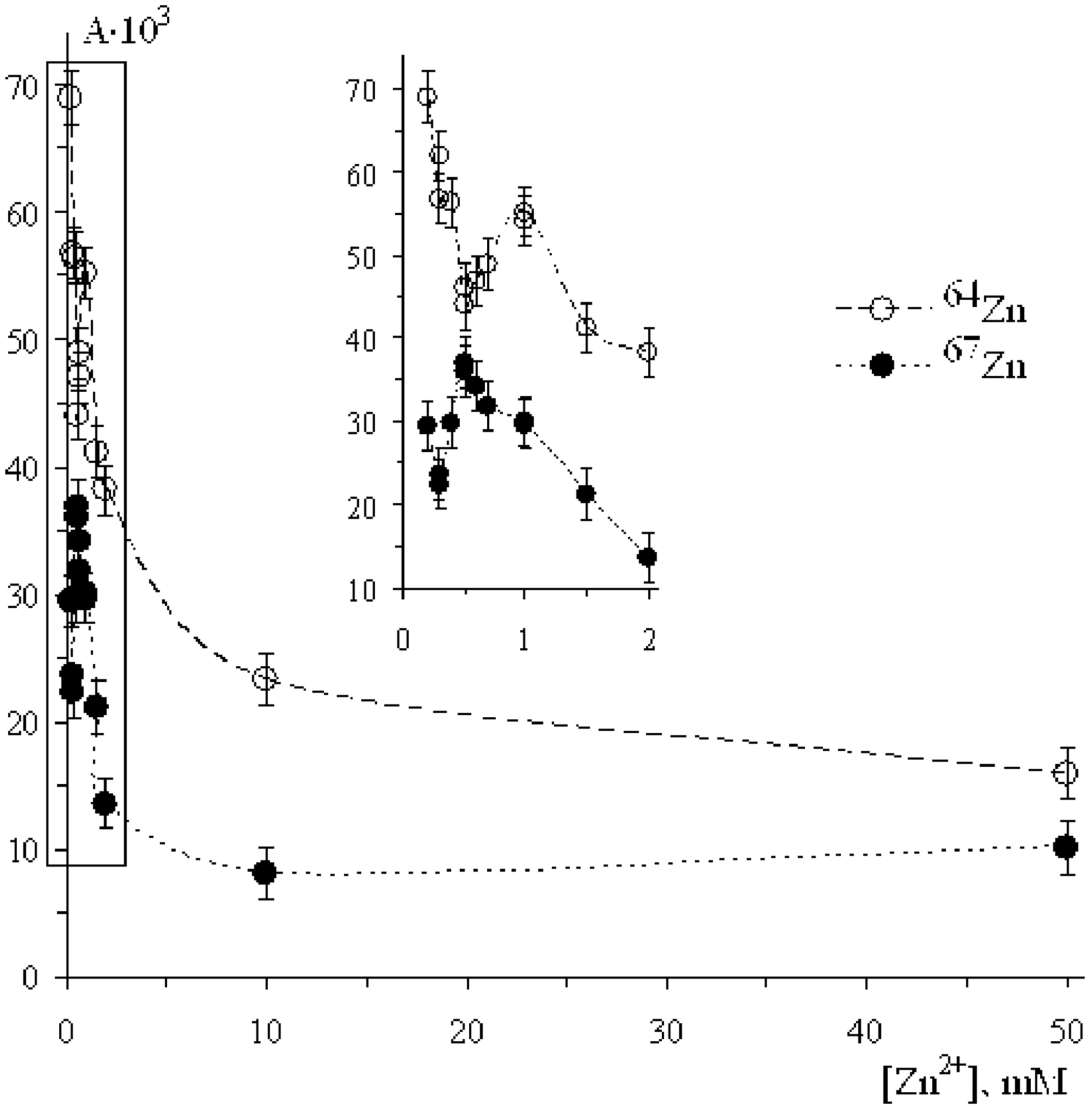
The rate of the DNA synthesis as a function of Zn^2+^ ion concentration. Tritium radioactivity A is measured as the number of counts/min/mg of DNA.

In these series of experiments, pol β was loaded either by Zn^2+^ ions with natural isotope composition (the content of magnetic isotope ^67^Zn in this sample is only 4.1% and further we will neglect it; the other Zn isotopes are spinless, non-magnetic) or by Zn^2+^ ions highly enriched with ^67^Zn (97.8%). These samples we will further signify as ^64^Zn and ^67^Zn, respectively. Figure 4 demonstrates concentration dependence of the pol β activity. It exhibits features very similar to those for magnesium ions. First, Zn^2+^ ions actually catalyse DNA synthesis with efficiency comparable with that of Mg^2+^ ions. Second, enzymatic activity of pol β with ^64^Zn^2+^ ions strongly exceeds activity of pol β with ^67^Zn^2+^ ions, i.e. magnetic Zn isotope suppresses pol β enzymatic activity. Third, the concentration dependence exhibits two maxima: one is expected to be in the range from zero to 0.5 mM, the second one is about 1 mM. Both maxima for ^67^Zn are shifted with respect to those for magnesium (Figures 1–3).

### Magnetic field effects

**Figure 5.**
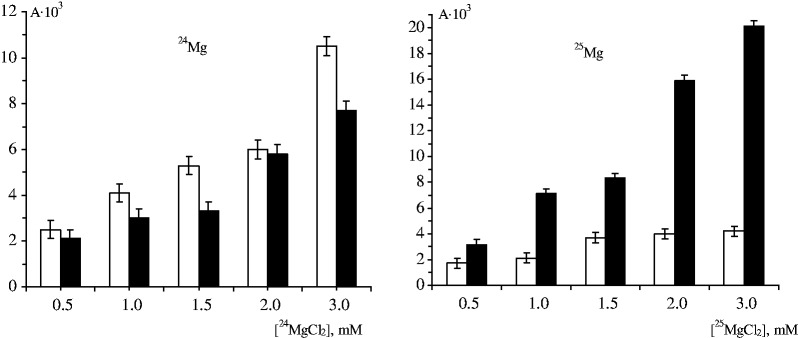
Magnetic field effect on the rate of DNA synthesis by pol β loaded with ^24^Mg^2+^ ions and with ^25^Mg^2+^ ions. Tritium radioactivity A is measured as the number of counts/min/mg DNA. Empty (left) columns refer to the experiments in the Earth magnetic field; black (right) columns refer to the experiments carried out in magnetic field 160 mT.

As seen in Figure 5, magnetic field effect on the DNA synthesis by pol β with ^24^Mg^2+^ ions is not too prominent; magnetic field only slightly suppresses DNA synthesis. The effect is systematic but does not strongly exceed the limits of experimental errors. However, it is clearly expressed with ^25^Mg^2+^ ions (Figure 5): at the concentrations of magnesium ions 0.5–3.0 mM, magnetic field increases the rate of the DNA synthesis by three to five times.

## DISCUSSION

The observation of magnetic isotope/magnetic field effects on the enzymatic DNA synthesis indicates that its mechanism includes paramagnetic intermediates and that besides of the nucleophilic mechanisms carefully analysed by Schlick *et al.* (6) there exists (and co-exists) another, ion–radical mechanism, similar to that which was recently discovered for enzymatic ATP synthesis (7,9).

### Ion–radical mechanism

**Scheme 1.**
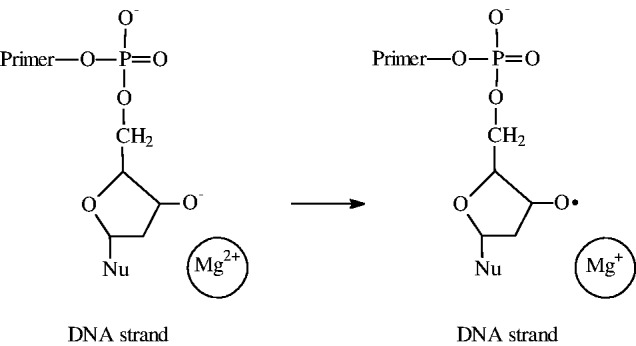
Electron transfer as a primary reaction of the DNA synthesis.

Nucleophilic mechanism of the nucleotide incorporation into the growing DNA macromolecule implies direct addition of 3′O^−^ ion of the ribose ring to the P_α_ atom of the incoming nucleotide and simultaneous release of pyrophosphate anion (1,2). Ion–radical mechanism suggests electron transfer from 3′O^−^ ion to the 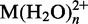 ion as a key, primary reaction (Scheme 1); it generates primary ion–radical pair composed of the oxy-radical on the ribose ring and ion-radical 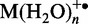 (M = Mg, Zn).

**Scheme 2.**
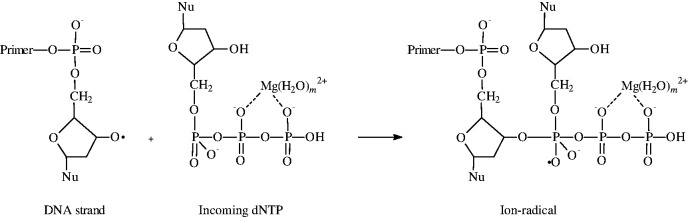
Ribose oxy-radical addition to the dNTP.

**Scheme 3.**
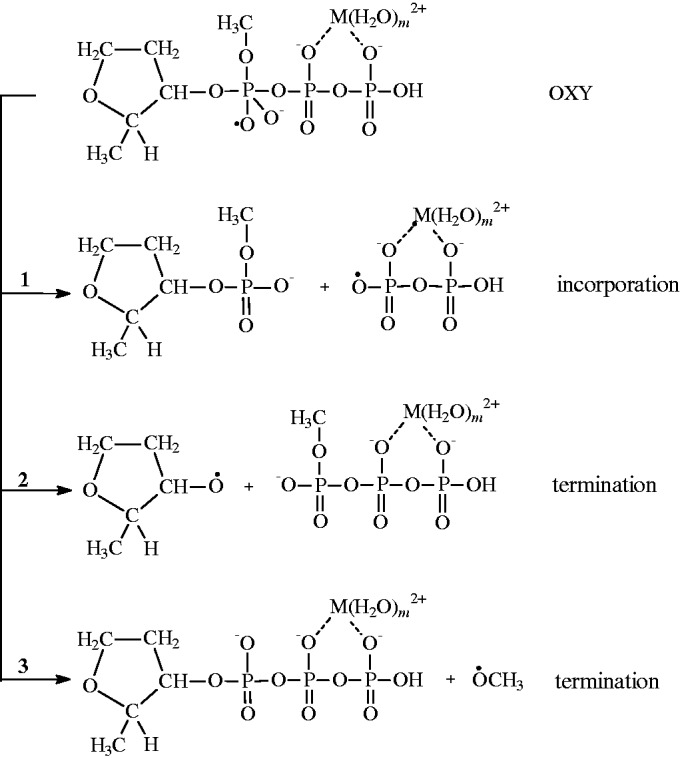
3 channels of the OXY decay (M is Mg or Zn).

The second step is an addition of the ribose oxy-radical to the P_α_–O double bond of the incoming nucleotide triphosphate dNTP (Scheme 2):

It generates a new oxy-radical (further it will be denoted as OXY), which decomposes by β-scission mechanism along the three channels (Scheme 3):

Only one of them, channel 1, results to the incorporation of nucleotide into the DNA molecule. The other two channels result either to the back reaction (channel 2) or to the destruction of nucleotide (channel 3). The latter two reactions are supposed to prevent (terminate) incorporation of nucleotide. Note that the final ion–radical pairs regenerate the starting 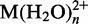 ions by the reverse electron transfer. For instance, in channel 1, this reaction generates pyrophosphate P_2_O_7_H^3^^−^

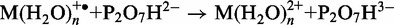

and regenerates the starting 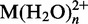 ion.

It is worthy to note that the ion–radical mechanism perfectly imitate nucleophilic one. The only difference is that instead of one-step nucleophilic reaction (addition of 3′O^−^ ion to the P_α_ atom of the incoming nucleotide synchronized with the release of pyrophosphate anion), ion–radical mechanism implies three steps: electron transfer, an addition of 3′O radical to the incoming nucleotide to generate new radical and the splitting of the latter to incorporate nucleotide into the growing DNA chain. From the concentration dependence of the DNA synthesis follows that the ion–radical mechanism, being also two-metal-ion mechanism, starts at approximately 0.5 mM; one can suspect that the nucleophilic mechanism may also function as a one-metal-ion reaction.

### Energies of the ion–radical reactions

**Figure 6.**
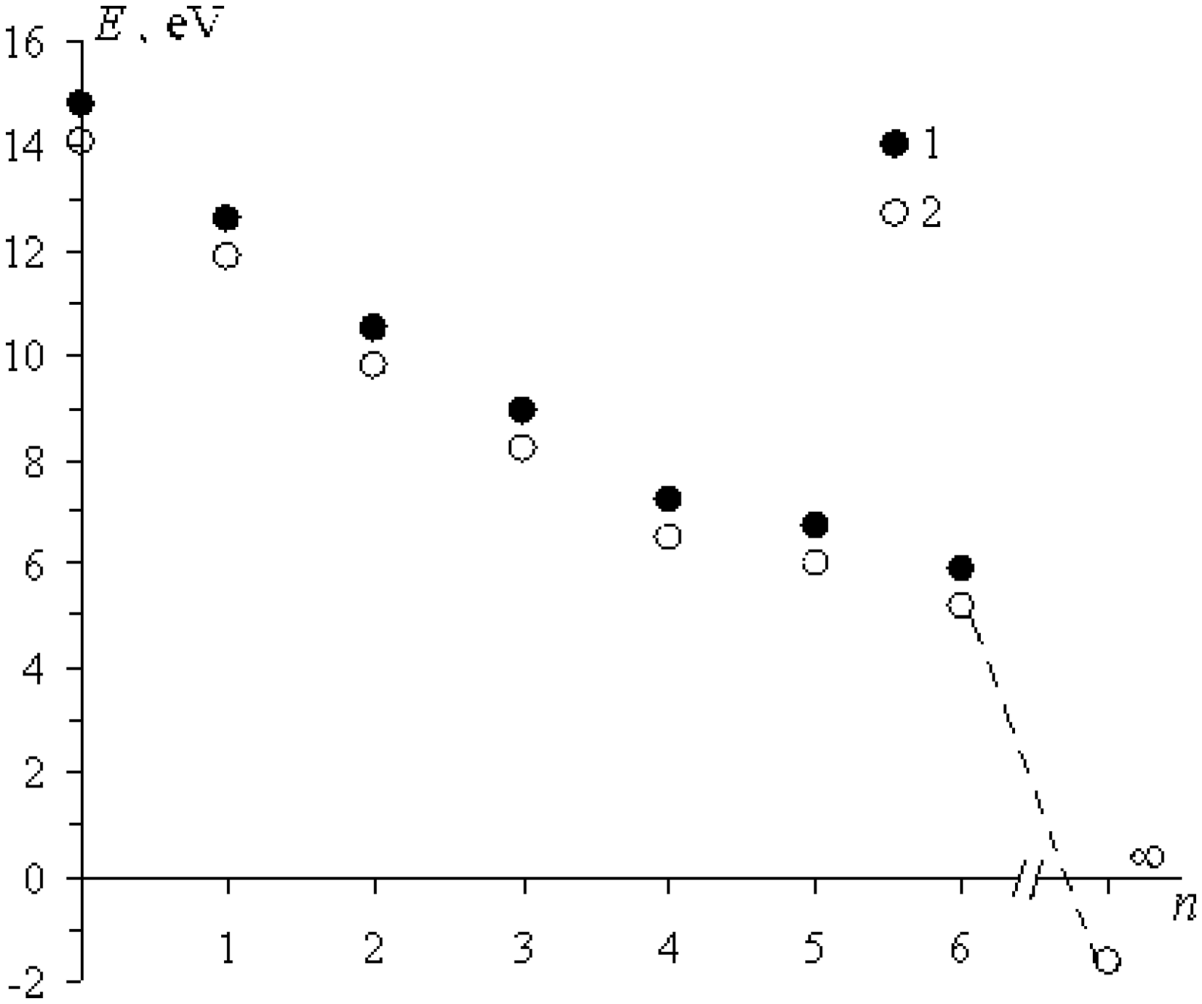
Energy of the electron transfer as a function of *n*, the number of water molecules in 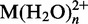 ions. M is Zn (1) and Mg (2). For *n* = 1–6 the energies were computed, for *n* = ∞ it is taken from (22).

Energies of the electron transfer were computed in terms of standard B3LYP DFT procedure (19–21) for the model reaction


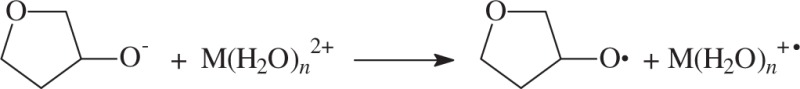


as a sum of two energies: the energy of electron detachment from the ribose oxy-anion and the energy of electron attachment to 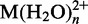 ion. Electron transfer is seen in Figure 6 to be exoergic, energy allowed process, even for ions with fully completed first hydrated sphere (*n* = 6); however, it is energy forbidden in water solutions (*n* = ∞). It is valid for 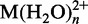 ions and is probably hold also for zinc ions.

One can assume that electron transfer in the catalytic site of pol β occurs near the thermally neutral boundary (*E* = 0). The energy barriers for direct and back electron transfer may fluctuate due to local fluctuations in hydrate shell of the ion (*n* may be more than 6). It means that both direct and back electron transfer (the latter regenerates starting reactants) may occur as a slightly exoergic, energy allowed processes.

The energy of the second step, the addition of the ribose oxy-radical to P_α_–O double bond, was computed to be almost independent on *m*, the number of water molecules in the coordination sphere of metal ions tightly bound with pyrophosphate residue (Scheme 2). The addition reaction is endoergic by 0.83 eV (Mg, *m* = 2) and 0.75 eV (Zn, *m* = 1, 2), so that the energies of radical and nucleophilic additions are comparable; the latter are in the range 0.52–0.93 eV (5).

The energies of the third step, decomposition of the secondary oxy-radical along the three channels (Scheme 3), are shown in Table 2. It demonstrates that both terminating channels 2 and 3 are exoergic while the incorporating channel 1 is endoergic and energy forbidden.

**Table 2.** The dissociation energies (in eV) of the oxy-radical OXY along the three channels

Ion	*m*	Channel 1	Channel 2	Channel 3
Mg^2+^	0	−1.92	−	+0.82
	1	−1.58	−	+1.01
	2	−1.50	+0.83	+0.71
Zn^2+^	1	−2.02	+0.75	+0.63
	2	−1.38	+0.75	+0.61

### Isotope and magnetic field control of the DNA synthesis

The source of the magnetic isotope/magnetic field effects is the primary ion–radical pair (22,23). As any pair thermally generated from the diamagnetic molecules, it is in a singlet spin state. Due to the spin allowed back electron transfer, the lifetime of the pair is strongly restricted, so that the next step, the addition of the oxy-radical to the P_α_–O double bond, is suppressed. However, if magnesium or zinc ions with magnetic nuclei ^25^Mg or ^67^Zn are presented in the catalytic site, then singlet–triplet spin сonversion, induced by hyperfine coupling in the ion-radicals 
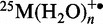
 or 
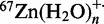
, transforms short-living singlet pair into the long-living triplet pair, in which back electron transfer is spin forbidden. The increased lifetime of the triplet pair makes the addition of the ribose oxy-radical to P_α_–O double bond more preferable. It generates secondary oxy-radical (Scheme 2), which produces nucleotide incorporation along the channel 1 with low efficiency because this channel is endoergic, in contrast to exoergic terminating channels 2 and 3. According to this mechanism, metal ions with magnetic nuclei stimulate singlet–triplet spin conversion and suppress DNA synthesis; this conclusion is in a perfect agreement with the experimental findings.

Spin conversion in the primary ion–radical pair is known to occur along the three channels: S–T_0_, S–T_+_, S–T_–_. In the pair [3′O• ^24^Mg^+^] only Zeeman interaction functions, which stimulates S–T_0_ transition; its rate is proportional to ΔgβH where Δg is a difference of g-factors of the radical pair partners, ribose oxy-radical and 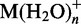 ion-radical. It accelerates singlet–triplet spin conversion and slightly suppresses DNA synthesis, in accordance with experiment (Figure 5, left).

In the pair [3′O• ^25^Mg^+^] hyperfine coupling with ^25^Mg nucleus dominates; it stimulates all three channels of spin conversion: S–T_0_, S–T_+_ and S–T_–_. However, magnetic field prevents singlet–triplet spin conversion along the two channels S–T_+_ and S–T_–_, i.e. it switches off these two channels induced exclusively only by hyperfine coupling. As a result, magnetic field in a great extent suppresses triplet channel of reaction, i.e. it functions opposite to the magnetic isotope effect stimulating DNA synthesis, in accordance with experiment (Figure 5, right). Moreover, as ΔgβH<*a*, where *a* is the hyperfine coupling constant in 
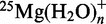
 ion, the contribution of Zeeman interaction is not too large in comparison with hyperfine coupling; this is a reason why magnetic field effect is less prominent with ^24^Mg ions than with ^25^Mg ions (Figure 5).

### Dependence of the pol β activity on the concentration of metal ions

No doubt that the first metal ion in the catalytic site is tightly bound with pyrophosphate residue. According to generally accepted concept, the entering of the second ion into the site switches on a standard two-magnesium-ion nucleophilic mechanism to attach incoming nucleotide to DNA strand. It is accompanied by enhancement of the pol β catalytic activity and simultaneously by appearance of the isotope effect. The latter unambiguously indicates that the ion–radical pathway of the DNA synthesis, competitive with nucleophilic one, is switched on; moreover, it further becomes dominating. However, the successive entering of the next ions at first slightly suppresses catalytic activity, but then strongly enhances both catalytic activity and isotope effect (see Figures 1–4). Such behaviour is enigmatic; nevertheless, one can suggest that the excess of metal ions may lower energy of pyrophosphate departure and direct reaction along the ion–radical channel 1 making it exoergic.

The intracellular concentration of metal ions is rather low, so that the dominating mechanism of the DNA synthesis in living organisms is thought to be a nucleophilic reaction. However, the contribution of the ion–radical channel of DNA synthesis in living organisms may appear to be significant or, at least, not ignorable, because it functions even at low level of magnesium ion concentration close to physiological one, i.e. at 0.5 mM and even less; moreover, it may depend on the type of polymerase.

### Critical comments

Ion–radical mechanism of the DNA synthesis seems to be unbelievable; it has nothing to do with commonly accepted nucleophilic mechanism. First idea which occurs in mind is that of an artifact. However, both isotope and magnetic field effects give direct and reliable evidence of its validity. We are perfectly aware that these effects as the physical phenomena are understandable in terms of Zeeman interaction and hyperfine coupling in the ion–radical pairs, paramagnetic intermediates on the reaction trajectory of the nucleotide incorporation into the DNA strand (24,25). However, in contrast to physics, chemistry of this reaction is far from being unambiguous and clear. We have considered and analysed almost dozen feasible reaction schemes, but no one of them was adequate to describe DNA synthesis and magnetic effects simultaneously, for exception of that proposed in this article.

Ion–radical mechanism satisfactorily explains the main properties of the reaction—isotope and magnetic field effects; however, there remains some questions unanswered. The first is why there exist two maxima in the concentration dependence of the DNA synthesis. No doubts that the standard two-metal-ion mechanism (1,2) does not function at high concentrations of metal ions. The remarkable increase of the rate of DNA synthesis and isotope effect at the ion concentration of 5–15 mM can be hardly explained in terms of this mechanism. Many-metal-ion mechanism is expected to function at these conditions; however, its details remain enigmatic. No doubts that it has no importance in living organisms, but it may appear to be useful in medicine. To answer this as well as the other possible questions, further examinations are required. First of all, it would be desirable to more carefully investigate magnetic field dependence as a function of metal ion concentration; the other enzymes (DNA and RNA polymerases, telomerase, etc.) are worthy to be studied. This work is now in progress.

At last, we would like to point out that some ion–radical mechanisms of spin-dependent synthesis of DNA/RNA were suggested by Tulub et al. (26,27). Magnesium isotope effects on the growth of *Escherichia coli* cells as well as protective function of ^25^Mg ions in the post-radiation recovery of the cells of yeast *Saccharomyces cerevisiae* enriched with ^25^Mg ions were also detected (28–30).

## CONCLUSION

It is important to note that the discovery of magnetic effects on the DNA synthesis simultaneously proves classical, nucleophilic mechanism and discloses a new, ion–radical mechanism. Indeed, at low concentrations of metal ions, there are no magnetic effects (see Figure 1–4; by the way, this is one of the arguments refuting idea of artifacts); they appear only at the elevated concentrations. Intracellular concentration of metal ions is rather low so that in living organisms the dominating mechanism of DNA synthesis is assumed to be a nucleophilic reaction; ion–radical mechanism functions under conditions when the concentration of metal ions is higher. Perhaps, both mechanisms, nucleophilic and ion-radical, coexist and function independently with the relative contributions depending on the concentration of metal ions: at low magnesium concentrations a nucleophilic mechanism operates; at high concentrations, on the contrary, an excess of catalytic ions switches ion–radical mechanism on. At the intermediate concentrations, both mechanisms are thought to coexist and function independently.

Despite the fact that there is yet no detailed understanding of the ion–radical mechanism, we are perfectly aware that the discovery of magnetic effects on this very important biochemical process may be used for medical purposes. Isotopic and magnetic field control of the DNA synthesis is thought to be a phenomenon of promising biomedical importance. In particular, the remarkable medicinal effects in the famous and widely used technologies of trans-cranial magnetic stimulation may be enhanced by influence of magnetic isotopes and magnetic fields on the efficiency of the DNA replication and gene expression. Magnetic control of the DNA replication may be used for other biomedical purposes (to kill cancer cells, to control cell proliferation, etc.).

## SUPPLEMENTARY DATA

Supplementary Data are available at NAR Online, including [31–42].

Supplementary Data
