# Supplementary material for: Magnetic isotope and magnetic field effects on the DNA synthesis
Source: Nucleic Acids Res. 2013 Jul 13;41(17):8300–7. doi: 10.1093/nar/gkt537 (PMC3783157; doi:10.1093/nar/gkt537)
Supplement: Supplementary Data [file supp_41_17_8300__index.html]

Magnetic isotope and magnetic field effects on the DNA synthesis — Supplementary Data 

# Magnetic isotope and magnetic field effects on the DNA synthesis

## 

files

**Files in this Data Supplement:**

- Supplementary Data - doc file
