# Supplementary material for: Magnetic isotope and magnetic field effects on the DNA synthesis
Source: Nucleic Acids Res. 2013 Jul 13;41(17):8300–7. doi: 10.1093/nar/gkt537 (PMC3783157; doi:10.1093/nar/gkt537)
Supplement: Supplementary Data [file supp_gkt537_nar-03306-f-2012-File001.doc]

Supporting Materials

“**Magnetic Isotope and Magnetic Field Effects on the DNA Replication”**

Anatoly Buchachenko,^*,†⁫^, Alexei Orlov **^§^**, Dmitry Kuznetsov^†^,**^§^**

The DFT calculations of the oxy-radical and its dissociation products were performed for the simplified structures with the following replacements. First, the fragment

in the ribose group of DNA strand was replaced by CH_3_ group; second, fragment

in the incoming nucleotide was replaced by −OCH_3_ group. These substitutions are through to hardly distort the energies of the radical dissociation taking into account that these energies are computed as the differences between those of reaction products and starting oxy-radical.

Three channels of radical splitting are shown below:

Only one of them, channel 1, results to the incorporation of the incoming nucleotide into the DNA strand. It is accompanied by release of pyrophosphate group and facilitates DNA replication. The other two channels result to the escape of nucleotide (back reaction, channel 2) or its destruction (channel 3); they terminate DNA replication.

The energies of the radical transformation along these three channels are presented in Table 0. Both terminating channels, 2 and 3, are seen to be exoergic by almost 1 eV for Mg and Zn ions. In contrast, the incorporating channel 1 is endoergic by 1.5-2.0 eV. It immediately results to conclusion that in the long-lived secondary triplet pair oxy-radical OXY predominantly undergoes decay without incorporation. For these reason magnetic isotopes of Mg and Zn, stimulating singlet-triplet spin conversion, populate triplet states of the pair and suppress incorporation of dNTP and DNA replication.

Table S0. Dissociation energies of the oxy-radical OXY along the three channels (in eV).

| Ion | *m* | Channel 1 | Channel 2 | Channel 3 |
| --- | --- | --- | --- | --- |
| Mg^2+^ | 0 | -1.92 | − | +0.82 |
|  | 1 | -1.58 | − | +1.01 |
|  | 2 | -1.50 | +0.83 | +0.71 |
| Zn^2+^ | 1 | -2.02 | +0.75 | +0.63 |
|  | 2 | -1.38 | +0.75 | +0.61 |

DNA β-POLYMERASE FROM HUMAN MYELOID LEUKEMIA CELLS

The HL-60 human myeloid leukemia cell line has been purchased from the Hungarian Cell Bank, Pasteur Institute of Hungary, Szeged, NCBI Code C427. Cells were maintained in suspension culture at +37°C under 5% CO_2_/air in RPMI 1640 (Gibco, UK) supplemented with 10% FCS and antibiotics: 100 U/mL Penicillin and 100 µg/mL Streptomycin. The cells were subcultured three times weekly, ATRA (Sigma, USA). This procedure has been originally adopted by [1] and then modified by [2]. The cells were precipitated by centrifugation at 12,000 rpm for 20 min (+4^0^C). The resulting pellets were then suspended and homogenized in 5 volumes of 20 mM MgCl_2_, using the glass-teflon Potter-Elvehjem homogenizer with a snug-fitting pestle (1,800 rpm).

Homogenates were filtered through the 5-layer cotton graze and then the filtrates were centrifuged at 800 g for 30 min (+4^0^C). The pellets were collected and re-suspended in 10 volumes of 15 mM Tris-HCl (pH 8.0)/1.5 mM EDTA/10 mM MgCl_2_/1.5mM KCl/25 mM sucrose/Trypsin 20 µg/mL (w/v) and incubated at +37°C for 1 hr with a following centrifugation at 800 g, 30 min (+4°C).

The crude nuclei fraction (P0.8) were homogenized in 5 volumes of 15 mM Tris-HCl (pH 7.60)/2.0% Triton X-100 (v/v)/10 mM MgCl_2_/2.5 mM KCl/2.5 mM EDTA/0.02% gluthatione (Merck, Germany)/0.05 heparin (Serva Heidelberg, Germany), 18,000 rpm, +4°C. Then the homogenates were simultaneously treated with nuclease S (80-100 U/mL, Worthington, USA) and pancreatic RNase A (50 µg/mL, Fluka, Switzerland), 60min, +37°C. Homogenates were then centrifuged at 150,000 g for 2 hrs (+4°C) in SW27.1 rotor, Spinco L5-75B Ultracentrifuge (Beckman, Austria). Pellets were extensively suspended in 6-7 volumes (w/v) of 25 mM Tris-HCl (pH 8.0)/3.0 mM EDTA/5.0 mM NaCl/1.0% 2-mercaptoethanol (v/v) at 50°C, 1 hr, with a subsequent phenol-chloroform protein extraction. To manage the latter, the basic mixture (phenol: chloroform, 1: 1, w/v) saturated with 20 mM Tris-HCl (pH 7.60) / 2.5 mM NaCl/1.5 mM EDTA/0.25% DMSO/1.0% 2-mercaptoethanol has been conventionally employed [3,4].

The resulting DNA/RNA-depleted phases were separated carefully and then mixed with 10 volumes of an ice-cold pure acetone. Then the samples were kept at +4°C overnight (10-12 hrs). All acetone – insoluble fractions were precipitated at 20,000 rpm, 20 min, +4°C. These pellets were extensively re-washed with acetone (3x – 4x) using the very same procedure.

Afterwards, the pellets were dissolved in 5-6 volumes (w/v) of 25 mM potassium-phosphate buffer (pH 6.30)/0.5% NaCl/1.5 mM EDTA/0.01% glutathione/0.05% heparin/1.0% 2-mercaptoethanol/80-100 U/mL nuclease S followed by 40 min incubation at +37°C. All post – incubation mixtures were treated by sonification at 80 KHz, 30 min, +60°C, under a non-stop extensive shaking.

Then the samples were submitted to a scalar fraction path reaching the 30% – 70% ammonium sulfate saturation, consequently. The precipitates obtained were collected at 10,000 rpm, 20 min, and dissolved in 15 mM potassium phosphate buffer (pH 6.0)/0.2% NaCl (10 vols, w/v). The solutions were subjected to dialysis against 20 mM potassium phosphate buffer (pH 6.0) to remove the excess of ammonium sulfate (+4°C, 12-16 hrs). The resulting samples were lyophilized for further use in a consequent gel filtration purification step.

The above specified technique is an original modification of a method described by [3] and developed by [4]. To prepare the onto-column applying samples, the lyophilized powders were first dissolved in 15 mM potassium phosphate buffer (pH 6.30)/5.0 mM MgCl_2_/1.5 mM EDTA/0.0001% sodium azide and passed through the fiberglass filters with 0.3-0.4 µ pore diameter (Millipore 5R, Moillipore, France). The transparent solutions were subjected to ultrafiltration on membranes with the molecular size exclusion limit of 25 kDa at 800 psi (Diaflo Y25, Amicon, Netherlands). The filtrates were adjusted with 2-mercaptoethanol up to 1.0% (v/v). The samples were concentrated in rotor evaporizer to reduce the final volume to about 1.5-2.5 mL.

The samples were applied onto the 1.5 × 50 cm (V = 98 mL) column packed with the TOYOPEARL HW 55F gel and equilibrated by the eluent buffer consisting of 15 mM potassium phosphate (pH 6.30)/5.0 mM MgCl_2_/0.0001% sodium azide. Elution rate: 0.8 mL/min (room temperature). UV-detector to scan at 280 nm has been employed. In each one of the consequently eluted 3.0 mL fractions, The DNA polymerase activity has been measured according to [5,6]. To get the enzyme specific activity measure (TTP-DNA incorporation rate per one milligram of protein used), the amounts of protein were estimated by the Bradford colometric method [7]. A column was calibrated with a standard protein markers set to cover the following molecular mass values range: 12.5 kDa (cytochrome C) – 24.0 kDa (trypsine) – 45 kDa (ovalbumin) – 70 kDa (HSA) – 145.0 kDa (L-asparaginase). Each one of the markers were passed through the column separately. All markers were purchased from Serva Heidelberg GmbH (Germany). To detect the Ve value, a blue dextran 2000 (2,000 kDA) has been used.

A slightly modified Laemmli method [8] has been employed to estimate both molecular mass and purification extent of the DNA polymerase isolated. The peculiar fractionation parameters: 10% PAAG separation system based on a standard Tris-glycine (pH 8.30)/0.25% SDS, 0.1 × x 100 × x 100 mm slab gels, 180 V/gel (4.5 mA/cm). Calibration of the slabs was conventionally taken care of with a standard QR 460/5 protein marker kit covering the 12.5-120.0 kDa range (Miles Laboratories, USA).

A routine 2.0%-agarose gel electrophoretic technique has been employed [9]. For the nascent single strand DNA chain length estimation, the 150*n*-300*n* ranged poly (dT) markers were applied (Calbiochem-Novabiochem International, USA).

*Pure Enzyme Isoelectric Focusing*

For the purified enzyme pI characterization, two mutually supporting isoelectric focusing procedures were employed.

First, the descendent linear 10.0-3.0 pH gradient 1.0 x 80 x 80 mm PAAG slabs with a reported pI performance range of 9.6-3.5 (KomaBiotech, Korea) were used as originally described by [10] in modification [11].

In addition to the main IEF route, a cellophane – attached ultrathin 0.12 × 260 × 125 mm PAAG slabs with the 10.0-3.0 pH gradients (Clean Gel – IEF plates based on PAGE - Pharmalyte combination purchased from GE HealthCare Europe GmbH, Germany) were engaged according to [12]. In this case, the processed and unfixed gels were also used to trace-or-exclude a 3′,5′- exonuclease activity in the resulted DNApolβ-fraction [13].

1. Olins, A.L., Hermann, H., Lichter,P., Olins, D.E. (2000) Retinoic acid differentiation of HL-60 cells promotes cytoskeletal polarization. *Exp. Cell Res*., 254 (1): 130-142.

2. Roy, M.R., Thalang, V.N. Trakoontivakorn, G., Nakahara, K. (2004) Mechanism of mahanine-induced apoptosis in human leukemia cells (HL-60). *Biomed. Pharmacol*., 67: 41-51.

3. Voss, D.O., Plaut, G.W.E., Hagihara, H., Clendenin, J.S. (1967) Fractionation of chromatin compounds isolated from the Mammalian neoplastic cell nuclei. *Meth. Enzymol*., 10: 326 – 341.

4. Lerman, M. I., Abakumova, E.V., Podobed, O.B., Zlatopolsky, A.D. (1976) Isolation and study on the properties of the DNP- and RNP –particles from the Ehrich ascite carcinoma cells. In: *Advanced Methods in Biochemistry* (Orekhovich, V.N., Ed.), pp. 74 – 89, Meditsina Publ.:Moscow.

5. Matsumoto, Y., Kim, K. (1995) The nuclear DNA polymerases B: activity shifts and the DNA gaps beta-elimination control. *Science*, 269: 699 – 702.

6. Piersen, C.E., Prasad, R., Wilson, S.H., Lloyd, R.S. (1996) On the 5´,3´-Deoxynucleotidyl transferase catalytic activity expressed by the nuclear DNA polymerase B in Mammalian cells. *J. Biol. Chem*., 271: 1781-1785.

7. Bradford, M.M. (1976) An improved colorimetric technique for protein measurement. *Analyt. Biochem*., 72: 348 – 354.

8. Laemmli, U.L. (1970) An efficient polyacrylamide gel electrophoresis system for proteins separation. *Nature*, 227: 690 – 695.

10. Walker, J.M. (1994) Isoelectric focusing of proteins in polyacrylamide gels. In: *Methods in Molecular Biology. Series. B* (Walker, J.M., Ed.), vol. 32, pp. 59 – 65, Springer Verlag GmbH: Berlin - Heidelberg.

11. Katoh, R. (2011) *Analytic Techniques in Biochemistry and Molecular Biology.* Springer Verlag GmbH: Berlin-Heidelberg.

12. Gorg, A., Postel, W.& Westermeir, R. (1978) Ultrathin-layer isoelectric focusing in polyacrylamide gels on cellophane. *Analyt. Biochem*., 89: 60 – 70.

13. Rule, G.S. (1984) Quantitative assay of deoxyribonuclease activity after isoelectric focusing in polyacrylamide gels. *Analyt. Biochem*., 138: 99 – 106.

Table2.Tritium radioactivity A of DNA in presence of magnesium isotopes*

In contrast to enzymatic ATP synthesis, where triplet channel has stimulated ATP synthesis, in the DNA replication the switching on this channel suppresses incorporation of nucleotide into the growing DNA strand.
